# Supplementary material for: Metabolic and Structural Insights into Hydrogen Sulfide Mis-Regulation in Enterococcus faecalis
Source: Antioxidants (Basel). 2022 Aug 19;11(8):1607. doi: 10.3390/antiox11081607 (PMC9405070; doi:10.3390/antiox11081607)
Supplement: Supplementary file 1 [file antioxidants-11-01607-s001.zip › antioxidants-1833704 Supplementary.pdf]

SUPPLEMENTAL INFORMATION

Metabolic and structural insights into hydrogen sulfide mis-regulation in *Enterococcus faecalis*

Brenna J. C. Walsh[1], Sofia Soares Costa[2], Katherine A. Edmonds[1], Jonathan C. Trinidad[1], Federico Issoglio[2,3], José A. Brito[2]* and David P. Giedroc[1,4]*

[1]Department of Chemistry, Indiana University, Bloomington, IN 47405-7102 USA

[2]Instituto de Tecnologia Química e Biológica António Xavier, Universidade Nova de Lisboa, 2780-157 Oeiras, Portugal

[3]Instituto de Química Biológica de la Facultad de Ciencias Exactas y Naturales (IQUIBICEN) - CONICET and Departamento de Química Biológica, Universidad de Buenos Aires, Buenos Aires, Argentina

[4]Department of Molecular and Cellular Biochemistry, Indiana University, Bloomington, IN 47405 USA

This file contains Supplemental Tables S1-S3, Supplemental Movie M1, Supplemental Figures S1-S9 and Supplemental References

Supplemental Table S1. This is an Excel file that lists all *E. faecalis* proteins detected in the label-free proteomics experiments (refer to Figure 1, main text). Those proteins commented upon in the text are highlighted in red.

Supplemental Table S2. This is an Excel file that identifies the cysteine residues of all persulfidated (*S*-sulfurated) proteins in the proteome of *E. faecalis* in the presence and absence of exogenous Na_2S (refers to Figure 2, main text). Those proteins commented upon in the text are highlighted in red.

Supplemental Table S3. Data collection, processing, refinement statistics and model quality parameters for *Ef*CoAPR CoA-bound structure.

*Ef*CoAPR CoA-bound form - PDB entry 8A56		
Data Collection		
Synchrotron Facility	ESRF (Grenoble, France)	
Beamline	ID23-2	
Wavelength (Å)	0.873	
Data Processing		
	autoPROC/STARANISO	***autoPROC/AIMLESS***
Resolution range (Å)[a]	48.25 – 2.05 (2.26 – 2.05)	45.73 – 2.52 (2.57-2.52)
Space group	$C\,2\,2\,2_1$	
Unit cell parameters<br>*a, b, c* (Å)<br>*α, β, Υ* (º)	139.80, 194.81, 91.46<br>90, 90, 90	
Total no. of reflections	229 727 (9 588)	186 084 (9 493)
No. of unique reflections	54 207 (2 710)	42 108 (2 113)
Multiplicity	4.2 (3.5)	4.4 (4.5)
Completeness (%)	-	
Spherical	68.7 (13.5)	99.3 (99.7)
Ellipsoidal	92.5 (65.3)	-
Mean I/σ(I)	7.2 (1.6)	8.8 (2.2)
R_{merge} (%) [b]	13.6 (81.8)	12.0 (65.1)
R_{meas} (%) [c]	15.5 (94.2)	13.7 (74.0)
R_{pim} (%) [d]	7.4 (45.7)	6.4 (34.4)
$CC_{1/2}$ (%) [e]	99.6 (64.9)	99.6 (82.7)
Refinement		
	autoPROC/STARANISO	***autoPROC/AIMLESS***
R_{work} (%) [f]	18.62 (29.16)	-

R_{free} (%) [g]	23.38 (37.11)	-
RMSD Bonds (Å) [h]	0.003	-
RMSD Angles (º) [h]	0.525	-
Number of atoms	-	-
Protein residues	1077	-
Non-hydrogen atoms	8619	-
Macromolecules	8181	
Ligands	318	-
Waters	244	-
Ramachandran plot	-	-
Most favoured (%)	96.63	
Outliers (%)	0.19	-
Rotamer outliers (%)	0.12	-
Clashscore [i]	1.77	-
Molprobity score [j]	0.96	-
Average *B*-factors ($Å^2$)	37.97	-
Protein	38.21	-
Ligands	33.71	-
Solvent	33.37	-

[a] Information in parenthesis refers to the last resolution shell.

[b] $R_{merge} = \sum_{hkl}\sum_i |I_i(hkl) - \overline{I(hkl)}| / \sum_{hkl}\sum_i I_i(hkl)$.

[c] $R_{meas} = \sum_{hkl}[N/(N-1)]^{\frac{1}{2}} \sum_i |I_i(hkl) - \overline{I(hkl)}\,| / \sum_{hkl}\sum_i I_i(hkl)$.

[d] $R_{p.i.m} = \sum_{hkl}[1/(N-1)]^{\frac{1}{2}} \sum_i |I_i(hkl) - \overline{I(hkl)}\,| / \sum_{hkl}\sum_i I_i(hkl)$.

[e] $CC_{1/2}$ as described in ref [1].

[f] $R_{work} = \sum_h\sum_k\sum_l \frac{\{||F_o(h,k,l)|-|F_c(h,k,l)||\}}{\sum_h\sum_k\sum_l|F_o(h,k,l)|}$, where F_o and F_c are the observed and calculated structure factors for reflection h, respectively.

[g] R_{free} was calculated the same way as R_{work} but using only 5% of the reflections which were selected randomly and omitted from refinement.

[h] RMSD, root mean square deviation.

[i] Clashscore is the number of unfavorable all-atom steric overlaps ≥ 0.4Å per 1000 atoms [2].

[j] *MolProbity* score provides a single number that represents the central *MolProbity* protein quality statistics; it is a log-weighted combination of clashscore, Ramachandran not favored and bad side-chain rotamers, giving one number that reflects the crystallographic resolution at which those values would be expected.

Supplemental Video S1. Representation of the replica 1 MD simulation illustrating the trajectory of the bound coenzyme A from an "extended" to a "bent" conformation, where the CoA S1 atom moves from its initial position close to the S^{γ} of C42 to the S^{γ} atom of C508'.

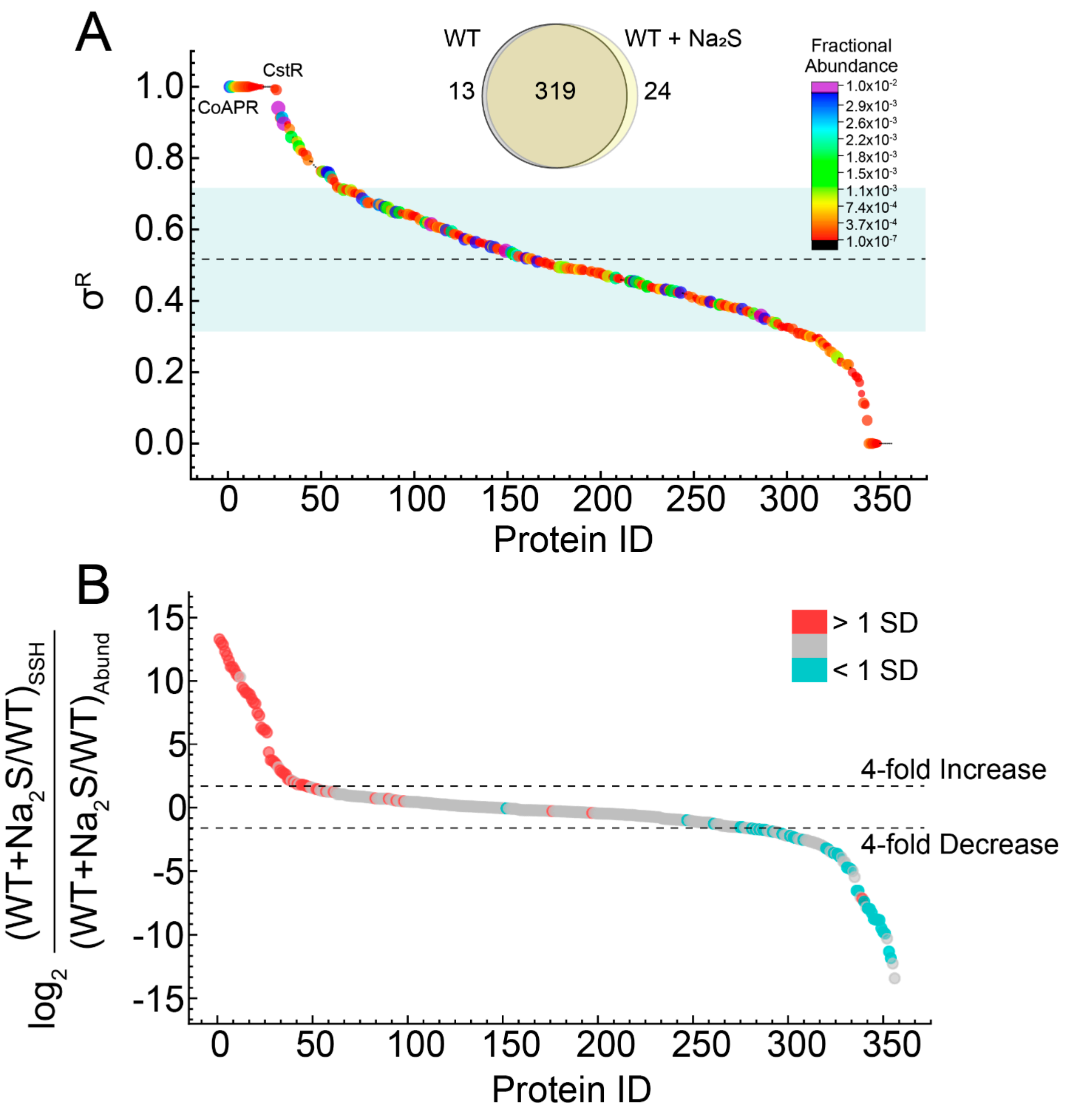

Supplemental Figure S1. Mapping proteome persulfidation in *E. faecalis* before and after addition of exogenous sodium sulfide. (A) Plot of σ^R versus protein ID, arbitrarily arranged from left to right according to σ^R (primary sort) and fractional abundance in WT+Na_2S (secondary sort). Each symbol represents a single protein and is colored and sized according to the fractional abundance of that protein in WT+Na_2S cells determined without enrichment. The dashed, horizontal line represents the mean σ^R (0.52), and the blue shaded area represents one standard deviation of the mean. σ^R is defined as the sum of all cysteine peptides in WT+Na_2S over the total cysteine peptides in both WT and WT+Na_2S, *i.e.*, $\Sigma(WT+Na_2S)/[\Sigma(WT+Na_2S)+\Sigma(WT)]$ [3]. (B) Proteome persulfidation normalized to protein abundance. Each symbol represents a single protein arbitrarily arranged from left to right with proteins color-coded according to σ^R with *red* being greater than ($\sigma^R \geq 0.75$) or blue representing less than ($\sigma^R \leq 0.29$) one standard deviation of the mean σ^R value (0.52). The dashed, horizontal lines represent a 4-fold increase or decrease in normalized protein persulfidation.

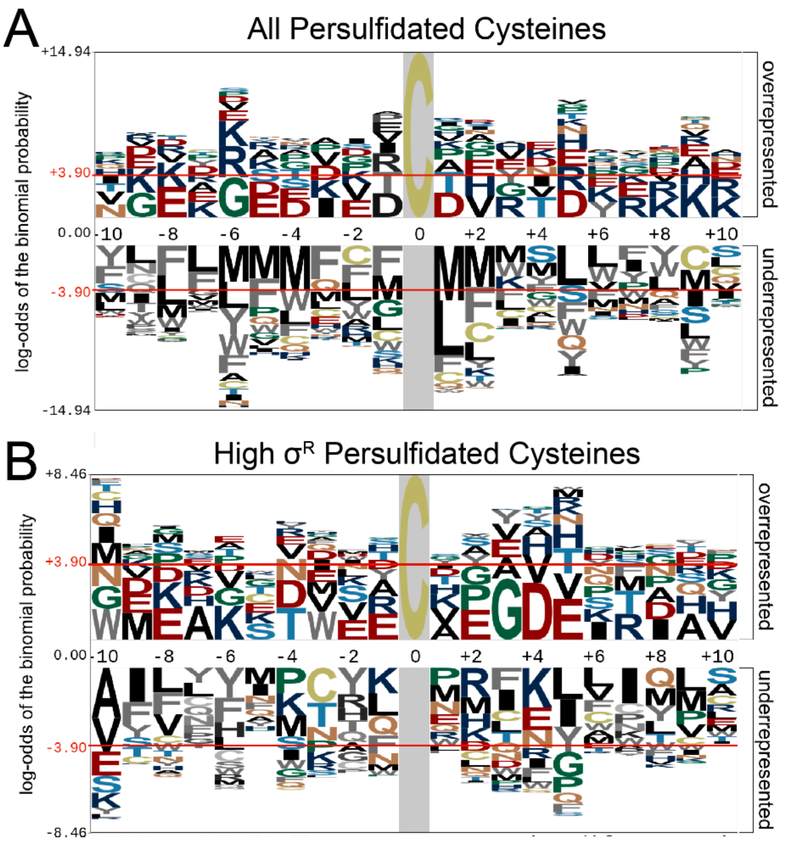

Supplemental Figure S2. Sequence motif analysis of *S*-sulfurated peptides. (A) Sequence motif analysis of persulfidation sites using pLogo [4] applied to all persulfidated cysteines identified in our data set relative to all cysteine containing proteins in the genome. The numbers of aligned foreground and background sequences are 490 and 4,792, respectively. (B) Same motif analysis applied only to high σ^R (above one standard deviation; not normalized to change in cellular abundance) sites. The number of foreground sequences is 85. For both, the red horizontal bars correspond to $P = 0.05$ and residues with a positive probability are overrepresented in the analysis while residues with a negative value are underrepresented.

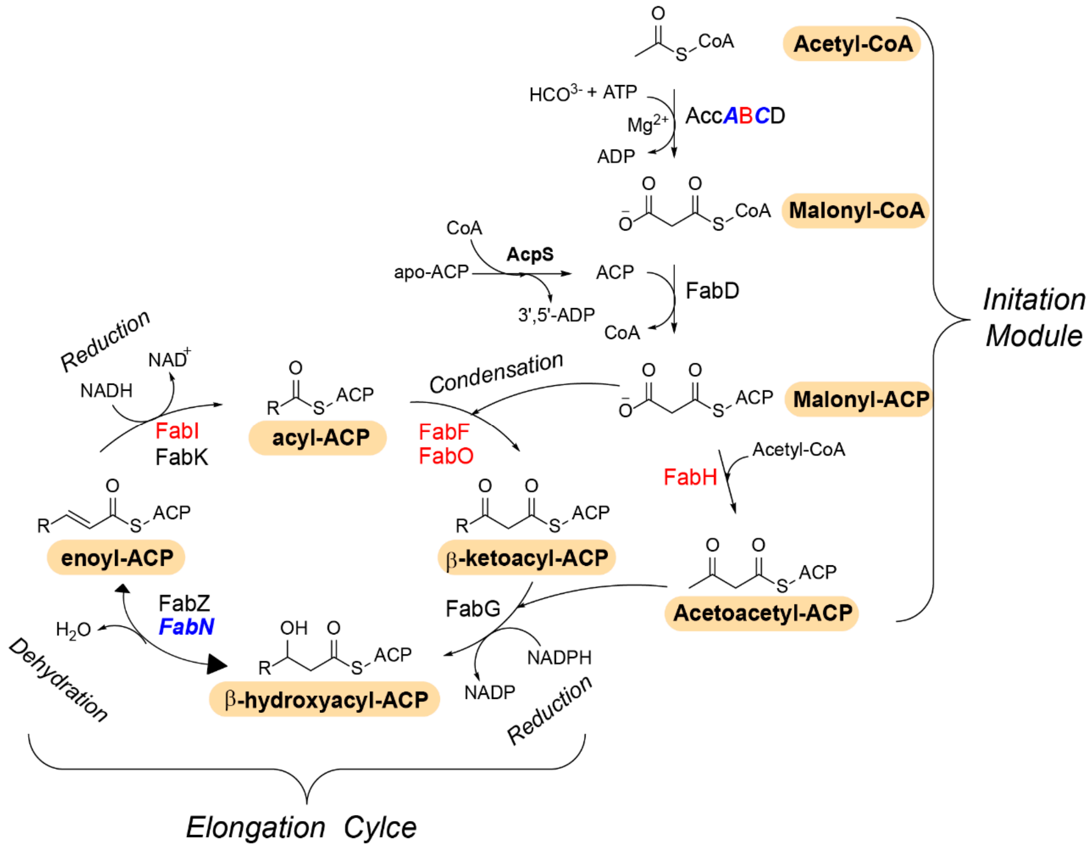

Supplemental Figure S3. Fatty acid biosynthesis in *E. faecalis.* Bacterial FAS adapted from Zhang *et al.* [5] consists of two modules, initiation, and elongation. Initiation requires acetyl-CoA to form malonyl-CoA by the AccABCD complex that is then transferred to acyl-carrier protein (ACP) followed by condensation with acetyl-CoA to form acetoacetyl-ACP that is then shuttled to the elongation module. Here, cycles of reduction, dehydration, and condensation reactions elongate the acyl-ACP by two carbon units each cycle. Note the equilibrium of the dehydratases (FabZ/N) lies largely to the β-hydroxyacyl-ACP as indicated by the arrow heads. **Bold**, *italicized* blue proteins indicated persulfidated proteins with significant changes in persulfidation status relative to their change in protein abundance and red proteins indicated the additional enzymes identified as persulfidated but not within the same significance threshold.

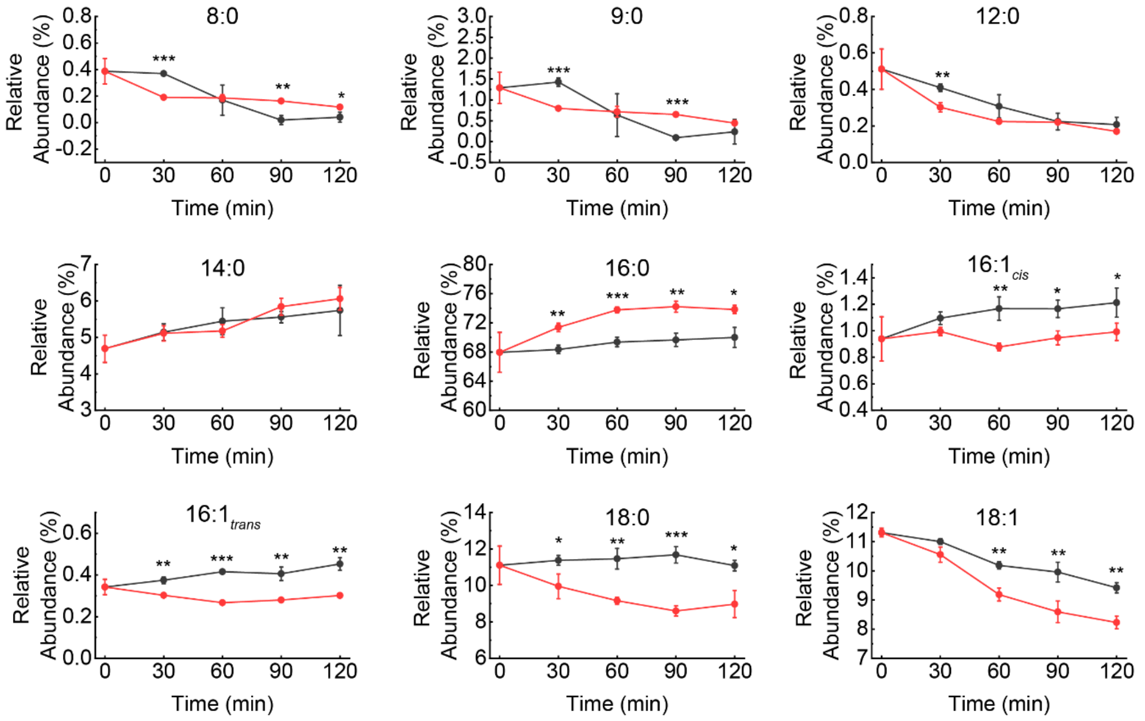

Supplemental Figure S4. Relative abundance of fatty acids before and after the addition of Na_2S. Changes in relative abundance for fatty acids extracted from wild-type (black) and Na_2S treated (red) cells. Values represent means ± S.D. derived from results of biological triplicate experiments with statistical significance established using a paired t-test relative to wild-type (***$p \leq 0.001$, **$p \leq 0.01$, *$p \leq 0.05$). Time 0 abundances are derived from n=6 replicates.

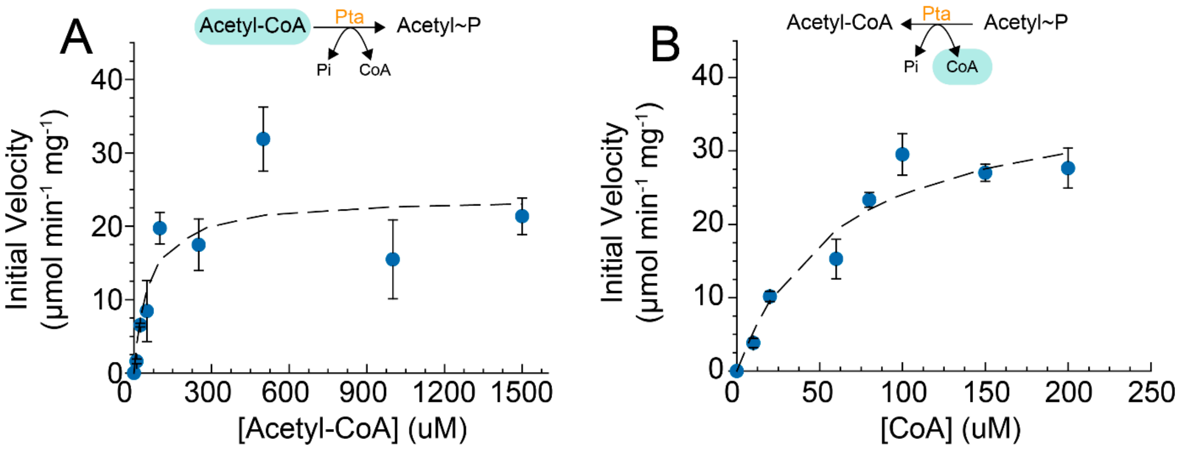

Supplemental Figure S5. Enzymatic activity of *E. faecalis* Pta. Initial velocity as a function of increasing (A) acetyl-CoA or (B) CoA substrate concentrations fit to a Michaelis-Menten model (dashed line). Values represent means ± S.D. derived from a triplicate experiment and fitted to the Michaelis-Menten equation (*dashed line*). Fitted parameters for panel A are K_m=57 (±16) µM and V_{max}=24.0 (±1.2) µmol min^{-1} mg^{-1} protein, yielding a k_{cat}/K_m of 146 (±34) mM^{-1} s^{-1} and panel B are K_m=69 (±14) µM and V_{max}=38.0 (±5.9) µmol min^{-1} mg^{-1} protein, yielding a k_{cat}/K_m of 360 (±60) mM^{-1} s^{-1}.

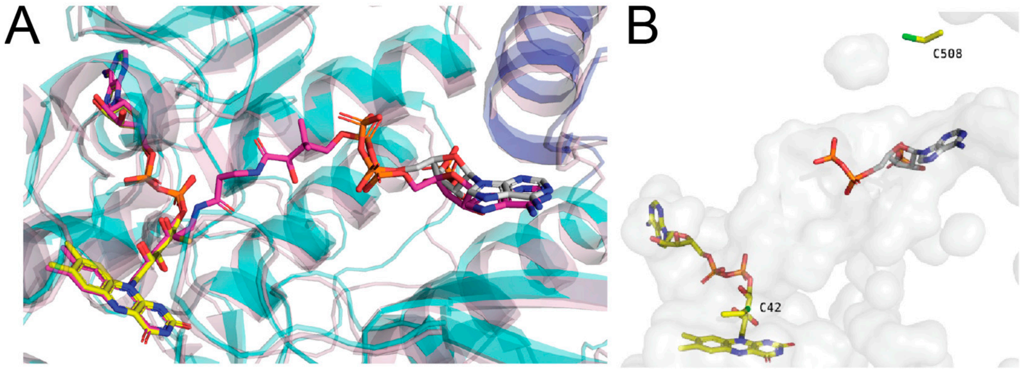

Supplemental Figure S6. Active site view and superposition of CoADR-RHD from *B. anthracis* (3ICS) [6] and *E. faecalis* CoAPR. (A) Cartoon representation of *Ef*CoAPR (shaded *cyan* for the CDR domain and *blue* for the rhodanese domain) and *Ba*CoADR-RHD (*light purple*). The nearly perfect superposition of the structures is evident, as well as the PAP moieties in both models. *Ef*CoAPR carbon atoms colored *yellow* for FAD and *white* for PAP. *Ba*CoADR-RHD carbon atoms colored *purple*. (B) Active site view showing a shallow cavity harboring the FAD, PAP, C42 and C508' depicted in surface mode. It is clear that there is sufficient space in this cavity for the missing pantothenate moiety of CoA in the *Ef*CoAPR structure, as well as for the movement for CoA to "swing" between C42 and C508' (from the opposite subunit).

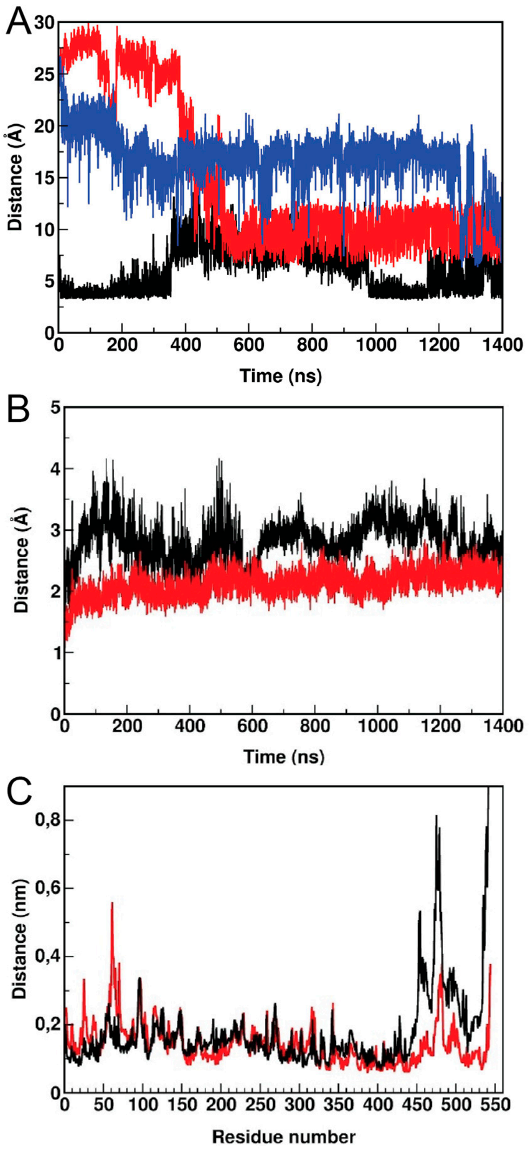

Supplemental Figure S7. Distance between the sulfhydryl group of CoA and each of the two cysteine residues C42 and C508' and a stability analysis of *Ef*CoAPR dimer during these MD simulations. (A) Analysis over two 1.4 μs simulation (replica 1, replica 2) of the distance between S1 of CoA and S^{γ} of C508 in one CoA binding site (*red* line) and between S1 of CoA and S^{γ} from C42 in the other CoA binding site in the homodimer in replica 1 (*black* line). For replica 2 the distance between S1 from CoA and S^{γ} from C508 is represented for the active site where the pantothenate arm is swinging (*blue* line). (B) Root mean square deviation (RMSD) of the backbone atoms in both subunits in replica 1. The protomer contributing the rhodanese domain to the active site where the CoA molecule is swinging *toward* C508 is colored in *red*, and the other subunit shown in *black*. (C) The difference in the displacement is related to the dynamics of the rhodanese domain (residues 447-544) and region of the CDR domain that packs against the rhodanese domain

(residues 58-73), as can be evidenced in the per-residue root mean square fluctuations. As in panel B, the protomer contributing the rhodanese domain to the active site where the CoA molecule is swinging *toward* C508 is colored in *red*, and the other subunit shown in *black*.

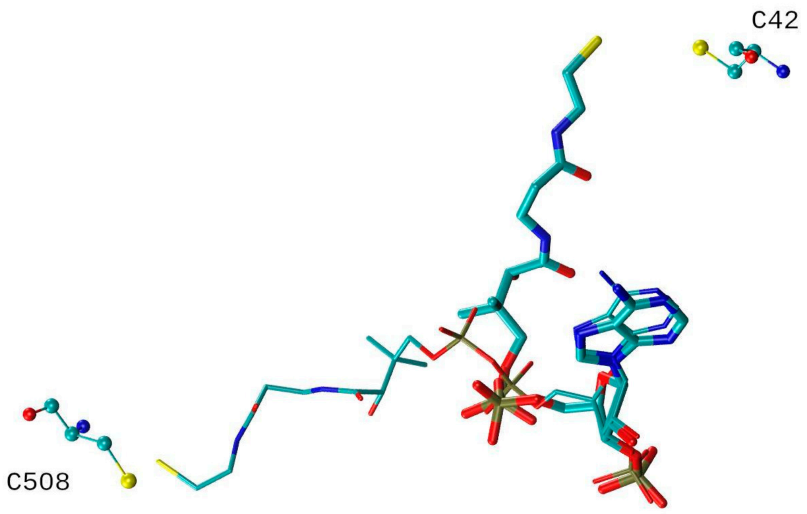

Supplemental Figure S8. Relative position of PAP and pantothenate moieties from CoA. Snapshots taken from replica 1 molecular dynamics simulations at the beginning (*thicker* sticks) and after reaching ≈6.5 Å distance between -SH group of CoA and -SH group of C508 (*thinner* sticks). C42 from one subunit and C508' from the adjacent subunit are depicted as spheres and sticks. See Supplemental Movie M1 for a trajectory of the switch.

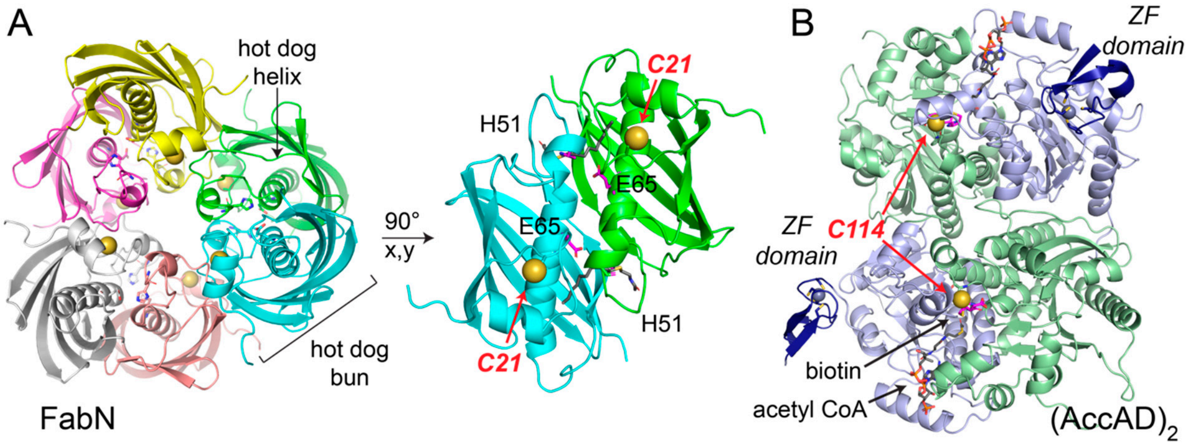

Supplemental Figure S9. Models of major persulfidation targets in *E. faecalis* under conditions of sulfide stress. (A) Ribbon representation of an AlphaFold2 [7,8] model of hexameric *Ef*FabN, illustrating the trimer of dimers architecture and the hot-dog fold of each monomer, with the hot dog helix cradled in a β-sheet bun (left panel). The sulfur atom of the persulfidated C21 is shown as a *yellow* sphere, while the active site residues H51 and E65 are shown as sticks. The right panel zooms in on a single dimer to illustrate the active sites with an

inhibitor modeled from PDB 1MKA (*gray* sticks). (B) Ribbon representation of an AlphaFold2 model of an *Ef*AccAD heterotetramer. Acetyl-CoA (*gray* sticks) and biotin (*magenta* sticks) bind in the active site cleft between the subunits (AccA, *green*; AccD, *light blue* with *dark blue* zinc fingers). C114 in AccA, with the sulfur atom shown as a yellow sphere, is highly persulfidated in cells, and is very close to the active site. Zn atoms are modeled as in PDB 1XNY, and biotin and acetyl CoA is modeled based on PDB 2F9I using Pymol (Version 2.4.1, Schrödinger, LLC).

Supplemental References

1. Karplus, P.A.; Diederichs, K. Linking crystallographic model and data quality. *Science* **2012**, *336*, 1030-1033.
2. Word, J.M.; Lovell, S.C.; LaBean, T.H.; Taylor, H.C.; Zalis, M.E.; Presley, B.K.; Richardson, J.S.; Richardson, D.C. Visualizing and quantifying molecular goodness-of-fit: small-probe contact dots with explicit hydrogen atoms. *J Mol Biol* **1999**, *285*, 1711-1733.
3. Walsh, B.J.; Giedroc, D.P. Proteomics Profiling of *S*-sulfurated Proteins in *Acinetobacter baumannii*. *Bio-Protocol* **2021**, *11*, e4000-e4000.
4. O'Shea, J.P.; Chou, M.F.; Quader, S.A.; Ryan, J.K.; Church, G.M.; Schwartz, D. pLogo: a probabilistic approach to visualizing sequence motifs. *Nat Methods* **2013**, *10*, 1211-1212.
5. Zhang, Y.M.; White, S.W.; Rock, C.O. Inhibiting bacterial fatty acid synthesis. *J Biol Chem* **2006**, *281*, 17541-17544.
6. Wallen, J.R.; Mallett, T.C.; Boles, W.; Parsonage, D.; Furdui, C.M.; Karplus, P.A.; Claiborne, A. Crystal structure and catalytic properties of *Bacillus anthracis* CoADR-RHD: implications for flavin-linked sulfur trafficking. *Biochemistry* **2009**, *48*, 9650-9667.
7. Jumper, J.; Evans, R.; Pritzel, A.; Green, T.; Figurnov, M.; Ronneberger, O.; Tunyasuvunakool, K.; Bates, R.; Zidek, A.; Potapenko, A.; et al. Highly accurate protein structure prediction with AlphaFold. *Nature* **2021**, *596*, 583-589.
8. Mirdita, M.; Ovchinnikov, S.; Steinegger, M. ColabFold - Making protein folding accessible to all. *bioRxiv* **2021** (doi: 10.1101/2021.08.15.456425).
